# THE FACTOR STRUCTURE OF MEMORY COMPLAINTS OVER TIME

**DOI:** 10.1093/geroni/igz038.808

**Published:** 2019-11-08

**Authors:** Jacqueline Mogle, Nikki Hill, Sakshi Bhargava, Tyler Bell, Emily Whitaker

**Affiliations:** Penn State University, University Park, Pennsylvania, United States

## Abstract

Although memory complaints are assessed with a variety of items to track change in individuals as they age, it remains unclear which items best capture change. Adults aged 70 to 104 (n=1,344, 38% Male) completed six memory complaints items annually for up to 11 years: frequency of problems, one year decline, ten year decline, seriousness of problems, forgetting important things, and current functioning compared to functioning at age 30. Using multilevel exploratory factor analysis, the best fitting model indicated one factor fit the between person structure with all items loading significantly. Across time, items required two factors. Items about decline loaded together while the item about functioning compared to functioning at age 30 dominated a second factor. Remaining items did not load on either factor across time. This suggests these items assessing memory complaints are better at discriminating across persons rather than tracking changes within a person across time.

